# The antioxidant components of milk and their role in processing, ripening, and storage: Functional food

**DOI:** 10.14202/vetworld.2019.12-33

**Published:** 2019-01-05

**Authors:** Imran Taj Khan, Mohammed Bule, Rahman Ullah, Muhammad Nadeem, Shafaq Asif, Kamal Niaz

**Affiliations:** 1 Department of Dairy Technology, University of Veterinary and Animal Sciences, Ravi Campus, Pattoki, Lahore-54000, Pakistan; 2 Department of Pharmacy, College of Medicine and Health Sciences, Ambo University, Ambo, Ethiopia; 3 Faculty of Veterinary Medicine, University of Teramo, Campus Coste Sant’Agostino, Renato Balzarini Street, 1, 64100 Teramo, Italy; 4 Department of Pharmacology and Toxicology, Cholistan University of Veterinary and Animal Sciences (CUVAS)-Bahawalpur-63100 Pakistan

**Keywords:** cholesterol, eggs, functional foods, milk, nutraceuticals, omega-3 polyunsaturated fatty acids

## Abstract

The current rate of population growth is so fast that, to feed this massive population, a 2-fold increase in land is required for the production of quality food. Improved dietary products such as milk and its products with antioxidant properties and functional foods of animal origin have been utilized to prevent chronic diseases. The designer milk contains low fat and less lactose, more protein, modified level of fatty acids, and desired amino acid profiles. The importance of milk and its products is due to the presence of protein, bioactive peptides, conjugated linoleic acid, omega-3 fatty acid, Vitamin D, selenium, and calcium. These constituents are present in milk product, play a key role in the physiological activities in human bodies, and act as anti-inflammatory, anti-tumor, antioxidant, hypocholesterolemic, immune boosting, and antimicrobial activities. Consumer awareness regarding benefits of designer foods such as milk and its products is almost non-existent worldwide and needs to be established to reach the benefits of designer food technologies in the near future. The main objective of this review was to collect data on the antioxidant properties of milk and its constituents which keep milk-derived products safe and preserved.

## Introduction

Milk is a rich source of nutrients and considered by many as a valuable element of a complete diet. Traditional mammalian milk such as cow’s milk contains many bioactive components that boost the physiological processes in the body. If global population continues to increase at the rate of 1.4%, then, by the year 2030, it will surpass 8 billion [1]. To feed this massive population, all countries need to increase their food production, which challenging owing to the shrinkage of free cultivable land and consumer awareness about designer foods health benefits via different nationwide programs [2]. Hence, there is a promising need to find new ways to produce nutritious food for this ever increasing population. Recent advancements in the field of biotechnology have brought hope for the production of adequate nutritious food [3].

Designer/functional food through biotechnological strategies promises quality food products that can be served to the people [4]. Designer food is genetically engineered food with enhanced nutritive value and is intended to improve the health of people. The term designer food seems sophisticated, but even the normal process of fermentation is a value addition to the food, and hence comes under the same domain [2]. Modification and manipulation of nutritive values of food derived from animal origin are of extreme interest due to the increasing demand for specialized market production [5]. Modifying the nutritional constituents of the diet supplied to animals seems to be a promising approach to improve the health-enhancing ingredients of animal products. Intervention at this level not only improves the nutritive quality of animal origin food but also changes the perception of the public regarding the foods from animal origin [6-8]. By feeding animals to create nutritionally modified food products or using techniques, i.e., cross-breeding, genetic engineering, and induced genetic mutation, the designer foods are produced to enhance or reduce the presence of particular nutrients and/or to improve them. Designer foods can be created with a sufficient amount of nutrients, which are considered beneficial for health. Several nutrients such as Vitamins C and E, β-carotene, amino acids, L-carnitine, and other phytochemicals have been identified to be very effective in preventing issues related to the heart and other vital organs, cancers, and cataract. Many food items have been created containing a large amount of these beneficial ingredients for the betterment of human health [9-15].

The current review focuses on antioxidant components of milk and their possible role in processing, ripening, and storage.

## Designing Functional Food

A functional food should possess the following properties: (i) It should be derived from a natural resource, (ii) it should be taken by the consumers as a regular diet, and (iii) it should be helpful in maintaining or improving the health of consumers [16]. To achieve a quality functional food from animal origin, a well-planned nutritional strategy has to be implemented on animals, to obtain a healthy food item for human use. To achieve this goal, we must take a number of steps such as collaborating the activities of laboratories, developing protocols and models for assessment of the bioaccessible nutrients and availability of bioactive components that can be used in studies on animals or humans, and ultimately contributing to the animal health on the global platform [17-19].

**Figure-1:**
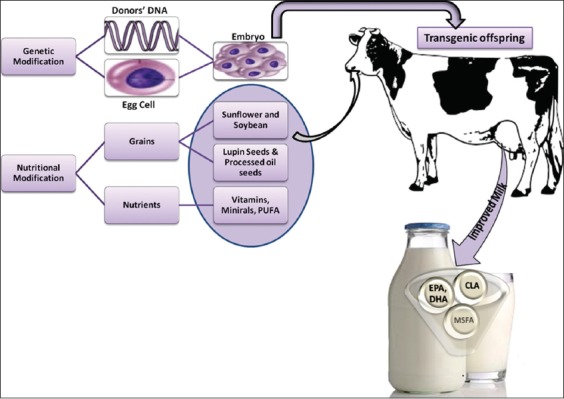
Designer milk with improved fatty acid and mineral content [Figure designed by Mohammed Bule].

Milk and its products possess several nutritive properties such as energy, protein, minerals, and vitamins [20]. Major reason behind the use of dairy products is to enhance the nutritive and dietary value of milk, subsequently for the betterment of human health as nutraceutical source [21]. The linoleic acid (LA) present in milk is known as the potential anticarcinogen, which can be controlled through diet management [22]. In recent years, it has been illustrated that casein micelle has the potential to transport polyphenols that show a significant decrease in colon cancer cells [23]. Thus, alterations in the diet of dairy animals could be focused to enhance the bioefficacy of bioactive polyphenols in milk to improve human health. As being more saturated, milk fat raises more public health concern than oils from plants. Hence, milk fatty acid composition needs to be improved by modifying the feeding strategies of the dairy animals [24]. A fatty acid profile of milk that has a great impact on oxidative stability of milk and dairy products has been mentioned for different species (cow, buffalo, goat, and sheep) as shown in Table-1 [25-28]. The role played by dairy nutrition in production of quality fat and fatty acids to increase the oxidative stability of milk has been studied worldwide [29-31]. Transfer efficiency is the term used to refer to the amount of fatty acids that can be transferred from dietary fat. Many studies illustrated that the transfer efficiency ranges from 2% to 4% for eicosapentaenoic acid (EPA) which is lower than docosapentaenoic acid (DHA) [32]. It was found that feeding fish oil in a rumen-protected manner increases transfer efficiency. Supplementation of 250 g/day fish oil produced 20-30% of transfer efficiency [33]. Diet is the major source of milk LA level, and many trials have been conducted with the aim to enhance milk conjugated LA (CLA) content (Figure-1) [21]. Increasing the rumen vaccenic acid output will raise the milk LA level [34]. Several vegetable oils have LA enhancing property in milk. Rumen biohydrogenation can be prevented by addition of oilseeds; hence, inclusion of oilseeds in the diet has been explored in many studies [35]. Many trials established that a high level of unsaturated fatty acid (UFA) in milk, particularly polyunsaturated fatty acids (PUFAs), increases the risk of oxidation and off-flavor [36]. A significant increase in milk monounsaturated fatty acids was found when the animals were fed with naked oats combined with grass silage or whole rapeseed grass [37]. Fish oil at recommended level in dairy diet seems to increase milk LA compared to vegetable oils. Combination of both fish and vegetable oils enrich with LA has the potential effect to increase milk LA level like soybean and sunflower [32,38]. Yu and Hu [39] found that, when cereals were substituted with lupin seeds, there was an improvement in milk production and higher concentration of milk protein and fat. Supplying sweet lupin to dairy cows considerably increased the milk C18:1 c-9 and reduced the saturated fatty acids (SFAs) compared to a control diet. Processed oil seeds enrich in LA and/or linolenic acid resulted to up regulate milk LA as compared to whole oilseeds, yet were not as fruitful as utilizing the pure oil [39]. Dietary supply of PUFA combined with modification to rumen environment can increase the milk fat LA. Feeding fresh pastures increases the milk CLA content by 2-3 folds [40]. Therefore, diet can extremely influence the levels of LA in milk fat, and there are likewise extensive contrasts between dairy species. The main reason for increasing milk fat LA is to increase the vaccenic acid outflow from rumen and increasing ∆9-desaturase activity. Through the diet and nutrition, the levels of LA can be improved; similarly, EPA and DHA can also be improved to a little extent by the above-mentioned way.

**Table-1:** Fatty acid profile of cow, buffalo, goat, and sheep milk.

Fatty acid	Cow (g/100 g of milk)	Buffalo (g/100 g of milk)	Goat (g/100 g of milk)	Sheep (g/100 g of milk)
C4:0	3.50	3.90	2.46	4.06
C6:0	2.30	2.33	2.40	2.78
C8:0	1.20	2.41	2.53	3.13
C10:0	2.60	2.40	9.38	4.97
C12:0	2.70	3.09	4.45	3.35
C14:0	9.30	10.64	10.16	10.16
C16:0	25.90	28.02	24.20	23.11
C18:0	14.30	12.58	12.51	12.88
*C18:1	27.60	24.10	23.01	26.01
*C18:2	2.10	2.04	2.72	1.61
*C18:3	0.70	0.68	0.53	0.92
References	[25]	[26]	[27]	[28]

* Fatty acids that have a significant impact on oxidative stability of milk and dairy products

## Genetic Modification (GM) of Livestock for Designer Foods

Genetically modified organisms are known as genetically engineered organisms, where genetic makeup has been changed by the addition of genes of interest from another living source, thereby tampering primarily to make animals more useful in respect of food production. Transgenic animals for the production of biological, therapeutics, and designer foods have reached the stage of commercialization [41,42]. The following approaches are used for the production of transgenic animals or animal products:

- Pronuclear microinjection: This technique is a well-standardized method used in several livestock [42]. This technique involves transfer of developmentally competent or transgenic embryos to donor females, thereby causing considerable reduction of cost [43,44].
- Embryonic stem (ES) cell manipulation: ES cell manipulation has been used mainly in mice for the deletion of target genes by homologous recombination to describe the function of specific genes but has not been used in other species [45]; however, ES-like cells have been used in pigs [46-48].
- Other alternative strategies: A prominent approach is transformation of animals using retroviruses [49].
- Nuclear transfer: In livestock, this sophisticated technique has been employed by the fusion of donor cell with an appropriate unfertilized egg or early embryo which has been enucleated [50].

Animals are usually subjected to GM for many purposes such as making them leaner with faster growth rates, requiring less food, and having special characteristics, such as increased milk production in dairy animals and growth performance in poultry [51,52]. These modifications again lead to improved productivity and lower costs for farmers and consumer, respectively. The U.S. Department of Agriculture and European Food Safety Authority are currently assessing various strategies of animal cloning for food products. Transgenic animals seem to be the next big step in cloning. However, it is not clear whether people will consider cloned animals as genetically modified foods [53].

Milk and its products produced from milk are healthy food nutrients containing sufficient amount of essential nutrients such as oleic acid, CLA, omega-3 fatty acids, short-chain and medium-chain fatty acids, vitamins, and minerals. Other bioactive compounds in milk and its food items may have positive health effects. However, little information is available regarding this very important and emerging aspect of nutrition [54]. Antioxidant capacity of milk and milk products is mainly due to sulfur-rich amino acids, such as tyrosine and cysteine (Table-2) [55,56], Vitamins A and E, carotenoids, and enzyme systems such as superoxide dismutase (SOD), catalase, and glutathione peroxidase (GSHPx) [57]. Milk also contains appreciable amounts of equol, a polyphenolic metabolite of daidzein, and antioxidant activity of this equol is scientifically well known [58]. Superoxide radicals (O_2_^−^), hydroxyl radicals, and peroxide radicals can be inhibited by the antioxidant systems of milk [59]. Dairy products constitute about 25-30% of the average diet of an individual [60]. Oxidation is fundamental to considerable living organisms for the production of energy necessary for all biological processes. Still, a greater risk to these biological systems is oxidative stress which can cause severe injury to them. It is illustrated that, when the oxygen is reduced, it produces a persistent amount of free radicals and reactive oxygen species (ROS) in the human body. Human body has numerous mechanisms for the neutralization and scavenging of ROS. The first line of defense against ROS is comprised of enzymes such as GSHPx, catalase, SOD, ubiquinol, and uric acid which also inhibit the excessive production of free radicals [61]. Intake of antioxidants in the form of supplements and foods rich in antioxidants may protect the body from oxidative stress and damage [62]. Lipid oxidation is the main reason for chemical spoilage in food and dairy products; it leads to objectionable changes in nutritional value, flavor, and texture of foods [63].

**Table-2:** Amino acids profile of cow, buffalo, sheep, and goat milk.

Amino acid (g/100 g proteins)	Cow	Goat	Buffalo	Sheep
Aspartic acid	7.80	7.40	7.13	6.50
Threonine	4.50	5.70	5.71	4.40
Serine	4.80	5.20	4.65	3.40
Glutamic acid	23.20	19.30	21.40	14.50
Proline	9.60	14.60	12.00	16.20
*Cystine	0.60	0.60	0.59	0.90
Glycine	1.80	2.10	1.93	3.50
Alanine	3.00	3.60	3.03	2.40
Valine	4.80	5.70	6.76	6.40
Methionine	1.80	3.50	0.92	2.70
Isoleucine	4.20	7.10	5.71	4.60
Leucine	8.70	8.20	9.79	9.90
*Tyrosine	4.50	4.80	3.85	3.80
Phenylalanine	4.80	6.00	4.71	4.30
Histidine	3.00	5.00	2.73	6.70
Lysine	8.10	8.20	7.49	7.80
References	[55]	[56]	[55]	[56]

* Amino acid in milk and dairy products that have antioxidant activity

### Designer milk and biological activities

The most bioactive constituents of milk are proteins such as immunoglobulins, lactoferrin, and peptides obtained from hydrolysis of proteins, and fatty acid component such as LA, some minerals, different oligosaccharides, and melatonin. Different methods of processing milk to have more desirable components besides a wide variety of on-farm management systems help in the enhancement of bioactive components that are found naturally in milk [64]. An overview on production of designer milk from transgenic offspring is depicted in Figure-1. Like eggs, milk can also be enriched with various nutrients such as vitamins and minerals. Transgenic techniques can be employed for the development of designer milk. Fortification of milk according to the consumer’s expectation can be done to strengthen the global demand of the milk [65]. Milk allergy is a common problem in children, so the removal of beta-lactoglobulin (β-LG) will eliminate this problem [66]. Genetic engineering can help in production of novel designer milk, thereby new milk products [42]. Stitched milk is designer milk that is rich in components of consumer choice, improving the consumers’ health by enhancing the immunity [67,68]. Milk is an ideal but complex food, capable of providing protection against infections, development of intestinal absorption, immune activation, reduce inflammation, anti-tumor, antimicrobial and promote establishment of unique gut micro-organisms [69]. Certain milk components provide multidirectional benefits. All oligosaccharides present in milk are able to control the entry of microbes by blocking the adhesion of pathogens on cell surfaces in addition to lactoferrin and lysozyme that can put forth direct antimicrobial effects. The colostrum provides passive immunity, which is capable enough to stimulate an increased host immune response. Understanding the functions of individual component of milk may help in formulation of a need-based fortified designer or transgenic milk.

Designer milk is manufactured by modification of primary casein structure or changing the lipid profile or increasing the protein recovery and adapting to the needs of infants. In addition to the presence of significant level of nutraceuticals, low milk fats change in the fatty acid profiles due to LA and omega-fats which have more beneficial activities [70], improved amino acid profiles, more varieties of proteins, less lactose to reduce calories value, and absence of β-LG to diminish milk allergy which are important considerations for modified milk production [71,72]. The ideal milk in terms of fat content should have >82% mono-SFAs, <8% SFAs, and <10% UFAs. Achieving this target is difficult though reduction in fat content can be explored [64]. Different approaches are used over a period of time for designing modified milk that will be accepted by the general public. Milk contains good quantity of fat for human, as one of its constituents, which are not appreciated by all people. Skimmed milk is a variety of milk, where the cream has been removed from milk to make it fat free. Several other approaches have also been tried to reduce the fat content of milk [73]. Abomasal administration of LA has reduced fat content of milk [74,75]. Rumen microbes can hydrogenate oils/oil seeds fed through oral route, and hence, they should be infused directly to abomasum and/or it should be given in an encapsulated form to pass rumen. Encapsulated canola oil feeding to cattle has increased UFA concentration in milk [76]. The milk obtained when used for butter making has good spreading compared to ordinary milk. Similar studies with encapsulation of both canola and soybean meal improved the butter spreading ability [76]. Focusing on enzymes such as acetyl-CoA carboxylase in mammary gland can reduce the synthesis of fat in milk [77]. Some researchers tried breeding policies to select animals which yield milk with less fat. Transgenic milk has been obtained from goats expressing various novel proteins and thereby enhancing the antimicrobial properties of goat’s milk. Transgenic milk with elevated lysozyme concentrations has successfully enhanced the development of the small intestine in goats, decreased the levels of inflammation, and reduced the colonization of enteropathogenic *Escherichia coli* in the intestine of neonatal and weaned pigs as well as in children [78]. Insomnia or sleep disorders can be treated with melatonin, and hence, enhancing the melatonin secretion from milk can help to treat insomnia [79,80]. As milk contains two major components; melatonin and tryptophan which help in sleep disorder and relaxation. This melatonin in the milk acts as intrinsic factor to treat sleeping disorder.

Goat milk is similar to human milk in composition. Humanization of milk means designing milk that resembles mother’s milk, which does not provoke allergic response in the infant babies. Manipulation should be aimed to alter protein, fat, and also oligosaccharides in the milk to achieve safe milk for the babies [65]. β-LG is present in cow’s milk but absent in human milk, and hence, it can elicit immune reaction when fed to infants [81,82]. Hence, infant formulation should lack this β-LG [83]. Another target may be incorporation of casein amino acid sequence of human into cow’s milk through gene manipulation. Lactoferrin and lysozyme should be incorporated into cow milk to increase the antibacterial properties of milk [65]. A variety of novel dairy fractions that could be included in infant formula has been identified, and the products are now commercially available. α-Lactalbumin is a compound with a very well-balanced amino acid profile useful in milk-based infant formulas. On breakdown into smaller peptides, it may exhibit various functions, such as enhancing mineral absorption, stimulating growth of beneficial microorganisms, modulating the immune system, and inhibiting the growth of pathogens [84]. Lactoferrin provides an assembly of bioactivities, including acceleration of iron absorption, initiation of intestinal proliferation and variation, regulation of immune function as well as antibacterial and antiviral effects [85]. The bovine osteopontin has many structural similarities to human lactoferrin, and it can affect bone mineralization and growth, making it a potential candidate protein for inclusion in infant formula [86]. Milk fat globule membranes have been shown to exhibit antimicrobial activities [87], and the presence of these components into complementary infants’ foods has been shown to lower the diarrhea incidence. Different constituents are fortified to milk and milk products to improve its quality, thereby its health benefits to human. Beverages based on milk enriched with apple and grape seed, prebiotic and probiotic fortified milk, folic acid-fortified milk, lutein-fortified milk, Vitamin D, calcium-fortified milk, and ice cream are already available for human consumption [88-92]. The use of natural products such as herbal extracts during processing of milk while preparation of cheese is reported recently [93].

## Oxidative Stress and its Consequences

During normal cellular and biochemical reactions, free radicals such as ROS and reactive nitrogen species (RNS) are generated which are usually counteracted by body’s own defense antioxidant system. However, in certain circumstances (diseases or exposure to certain toxic agents), either there is excessive production of these free radicals or the body’s antioxidant system weakens which leads to an imbalance. Oxidative stress is defined as the physiological imbalance between the production of free radicals and their neutralization by the antioxidant system [94]. Mitochondria are considered as a major source of free radicals or ROS generation where O^−2^ is formed by electron escape from complexes I and III of electron transport chain [95,96]. Furthermore, tumor necrotic factor-alpha, an inflammatory cytokine, stimulates the production of free radicals by the mitochondria. The most common environmental pollutants acting as an exogenous source of increasing oxidative stress are heavy metals, pesticides, benzene, and certain other chemicals (xylene, phthalates, and bisphenol) [97,98]. The resultant oxidative stress causes damage to the cell macromolecules such as DNA, proteins, and lipids and also disturbs signaling transduction inside cell.

### DNA damage

DNA damage has been seen in the form of oxidative DNA damage, DNA strands break, and mutations occur. Such alterations in DNA are related to numerous chronic diseases. Fragmentation of DNA base will yield several products, containing 8-hydroxyguanine (8-OHdG), thymine glycol, adenine, and hydroxyl methyl urea [99]. 8-OHdG is an altered nucleoside base, which is the most commonly detected by-product of DNA damage in many diseases such as cancer and diabetes and has been suggested as a sensitive biomarker for assessing oxidative stress level in diabetic patients [100,101].

### Lipid peroxidation (LPO)

LPO is the oxidative degradation of lipids by free radicals or ROS. In this scenario, free radicals snatch the electrons from lipid/fat molecules in cell membranes, and such chemical modification disrupts normal cell membrane functions by inactivating membrane-bound receptors and enzymes and also increases cell permeability. During this reaction, various aldehydes are formed. Malondialdehyde has been used for many years as a biomarker for assessing oxidative stress status in diabetes. Similarly, evidence exist about the elevated level of thiobarbituric acid reactive substances (TBARS) in diabetic patients. TBARS acts a biomarker for oxidative damage [102,103].

### Oxidation of proteins

Proteins are major targets to be effected in biological oxidation. They are susceptible to the action of ROS toxic effect. In addition to cell membrane component, proteins also constitute the enzymes which catalyze cellular reactions, so oxidative damage of proteins might have serious outcomes in certain diseases such as diabetes. The level of carbonyl esters can be employed as a biomarker of insulin resistance (IR) in type 2 diabetes, as it has shown a great correlation with homeostasis model assessment-IR and glycated hemoglobin. Carbonyl ester assays provide a quick means of measuring the oxidative stress status [104,105].

### Alteration of signal transduction

Apart from exerting toxic effects on cell macromolecules, ROS also can induce the expression of numerous genes involved in the signal transduction process [106].

## Antioxidant Properties of Milk

Antioxidants are chemical substances that can neutralize and scavenge the free radicals, which are continuously produced in the body [107]. Uncontrolled free radicals in body can lead to oxidative stresses, which have been implicated in the breakdown of vital biochemical compounds, lipids, protein, DNA, diabetes, accelerated aging, carcinogenesis, and cardiovascular diseases [108]. The anticipated carcinogenicity of synthetic antioxidants such as butylated hydroxyl anisole, butylated hydroxyl toluene, and tertiary butylatedhydroxyquinone has led to the increased usage of natural antioxidants for the stabilization of foods. Functional foods have several health-promoting substances beyond traditional nutrients. Change in lifestyle has a great deal of impact on disease patterns, and about 20-30 years ago, infectious diseases were more than non-communicable diseases, but now the non-communicable/metabolic diseases are on the higher side. In the current scenario, healthy/functional food should be selected to avoid and/or minimize non-communicable diseases, such as diabetes, cancers, and cardiovascular diseases [109]. Demand for food containing natural antioxidants is increasing across the globe. Large numbers of food and dairy products are being supplemented with natural antioxidants [110].

**Figure-2:**
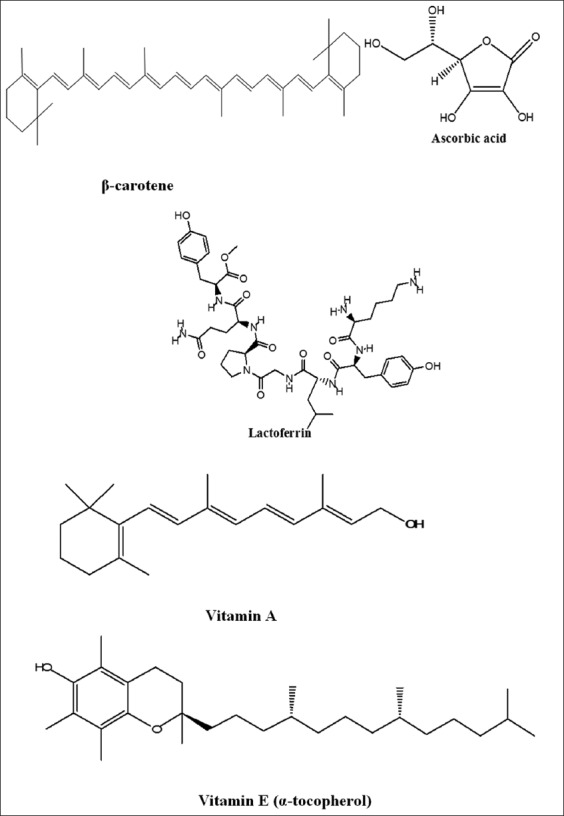
Major antioxidant molecules in milk [Figure designed by Mohammed Bule].

The leading antioxidants present in milk can be grouped into lipid-and water-soluble antioxidants. Carotenoids, retinol, and α-tocopherol are lipid-soluble antioxidants (Figure-2), whereas ascorbic acid is water-soluble antioxidant [111]. α-Tocopherol is largely present in milk fat globule membrane and it is considered the most effective lipid-soluble antioxidants present in milk [34]. It has properties such as preventative, chain-breaking antioxidant, and quencher of singlet oxygen in milk [60]. Milk can develop off-flavor as a result of photo-oxidation and contamination with copper, and existence of antioxidants in milk can inhibit the free radical mechanism by donating the proton and thus inhibit the onset of autoxidation. Vitamin E inhibits the activity of plasmin, a proteolytic enzyme, and second, it can directly scavenge the free radicals [112]. β-carotene is regarded as preventive antioxidant, it can also quench singlet oxygen, and one molecule of β-carotene can quench 250-1000 molecules of singlet oxygen [113]. Ascorbic acid or Vitamin C is the major and most important water-soluble antioxidant present in milk and has strong affinity to scavenge free radicals [114]. Ascorbic acid can scavenge superoxide, iron oxide, nitric oxide, and alkoxyl radicals [115]. Supplementation of 100 mg α-tocopherol/kg milk fat and 100 mg ascorbyl palmitate/kg milk fat to ultra-high-temperature milk decreased the concentration of hexanal during the storage period of 4 weeks [116]. Lactoferrin and casein can inhibit LPO, generation of peroxide radicals, TBARS, uptake of oxygen, and iron oxide free radicals [117]. Antioxidant activities such as SOD, catalase, GSHPx, casein, and certain peptides are well established [118]. GSH and selenium enhanced the functional value, antioxidant capacity of milk. Antioxidant activity of zinc and selenium for the inhibition of SOD is scientifically proven [119].

## Various Constituents of Milk and its Properties

### Antioxidant properties of caseins

Caseins are the major protein of bovine and ovine milk present in the form of macromolecular aggregates, and due to the difference in phosphate content, various casein fractions are present in milk (Table-3) [120,121]. For example, phosphate content of α, β, and κ caseins is 10, 5, and 1 moles per casein mole, respectively, while phosphates are responsible for providing the antioxidant activities on casein micelles. Antioxidation mechanisms of action are complex; however, higher effect was achieved by normalizing Fe2+/Fe3+; it seems that casein diminishes LPO due to autoxidation of iron [122]. Milk proteins have shown antioxidant capacity for the scavenging of ROS. Studies have shown that casein inhibited the lipoxygenase-catalyzed lipid autoxidation. Free amino acids cannot quench the free radicals, and for the scavenging of free radicals, primary structure of casein molecules acts as scavenger [123]. Phosphoserine residues associated with casein molecules and inorganic phosphate present in casein and serum can bind the non-heme iron. In a previous investigation, it was observed that 72% and 21% of the supplemented non-heme iron in skim milk were isolated from α- and β-caseins, respectively, phosphoryl-rich peptides of casein phosphatides can bind the divalent metal, and casein-derived peptides inhibited the lipoxygenase activity [124]. Casein-derived phosphopeptides revealed the primary and secondary metabolites responsible for the sequestering of iron and direct scavenging activity in lipid and aqueous food systems [125]. Browning is a serious problem in many foods, and casein-based coatings are commercially used to prevent oxidation-induced browning of fruits and vegetables. Efficacy of calcium caseinate and whey powder in delaying the enzymatic browning in slice potatoes and apples was investigated; the results showed that milk protein-based edible coating efficiently postponed the enzymatic browning. Whey protein powder revealed better antioxidant activity than calcium caseinate, and the differences in antioxidant activity of whey protein and caseinate were attributed due to the differences in amino acid profile [126].

**Table-3:** Casein fraction of cow, buffalo, sheep, and goat milk.

Parameters	Cow	Buffalo	Sheep	Goat
TPC (g/L)	27.8	49.2	59.4	33.4
αS1-Casein (%)	37	37	33	99
αS2-Casein (%)	7	17	14	8.52
β-Casein (%)	42	28	30	63
γ-Casein (%)	6	10	9	18
κ-Casein (%)	9	13	14	8
References	[120]	[121]	[120]	[120]

TPC=Total protein content

### Antioxidant properties of whey proteins

In recent years, utilization of whey in food and non-food applications is mounting across the globe. Whey protein has a higher biological value, although about 30-35% of the whey is still discarded [4]. In food industries, whey proteins are used as emulsifying, gelling, and bulking agent, and antioxidant activity of whey protein is scientifically established, and antioxidant of whey can efficiently inhibit the lipid oxidation [59]. Antioxidant activity of whey protein is due to the chelating transition metals by lactoferrin and sulfur-containing amino acid such as cysteine and tyrosine which scavenge the free radicals (Table-4) [120,127-129]. Whey boosts the level of GSH which is regarded as one of the most significant water-soluble antioxidants produced in the body [130]. Numerous studies have shown that whey proteins have antioxidant activity; addition of whey protein in soybean oil emulsion increased the oxidative stability [63]. Antioxidant characteristics of salmon oil emulsion increased as a function of addition of whey protein [122]. Fermented whey-based foods have better antioxidant activity.

**Table-4:** Composition of whey proteins in cow, buffalo, sheep, and goat milk.

Parameters	Cow	Buffalo	Sheep	Goat
Whey proteins (g/L)	6.46	6.46	10.76	6.14
β-Lactoglobulin (%)	59.30	59.30	61.10	54.20
α-Lactalbumin (%)	16.20	16.20	10.80	21.40
Immunoglobulins (%)	15.00	15.00	20.00	11.50
Serum albumin/lactoferrin (%)	9.50	9.50	8.10	12.80
References	[120]	[129]	[120]	[129]

### Antioxidant characteristics of carotenoids

Carotenoids are lipophilic molecules, and they have the tendency to accumulate in lipophilic region of cells such as membrane or lipoproteins [131]. As a feature of aerobic life, human body is exposed to a wide range of pro-oxidants that can cause serious damage to DNA, proteins, carbohydrates, and lipids. Among the various antioxidant systems in milk, carotenoids act a scavenger of singlet oxygen and peroxyl radicals [132]. One molecule of β-carotene can scavenge/quench up to 1000 molecules of singlet oxygen [133]. Dairy lipids may suffer from oxidation, which leads to a negative impact on quality and sensory characteristics of final dairy products. Autoxidation and light-triggered oxidation are mainly stimulated by a multifaceted interaction of pro- and anti-oxidants mechanisms. Vitamin E and carotenoids constitute fat-soluble antioxidants, and milk fat globule membrane is considered as the most volatile site for autoxidation [134]. Photo-oxidation is predominantly inhibited by β-carotene; it absorbs light that would otherwise be absorbed by riboflavin, which may give rise to quality-related issues. β-carotene absorbs light in a concentration-dependent manner [135]. Carotenoids act as scavengers of singlet oxygen and some ROS [134]. Results of an earlier investigation regarding the migration of carotenoids from milk to cheese and butter have shown that concentration of carotenoids was intensified in cheese and butter [136].

### Antioxidant characteristics of ascorbic acid and Vitamin E

Ascorbic acid is a vital, strong, and least toxic natural antioxidant, it is the main water-soluble antioxidant present in milk, and the free radical scavenging activity of ascorbic acid is due to low oxidation-reduction potential (330 mV). Ascorbic acid can scavenge superoxide anion radicals, alkoxyl radicals, and singlet oxygen [114]. Ascorbic acid significantly inhibited the degradation of riboflavin in cream in the presence of 1000 Lux light for 4 days [137]. Ascorbic acid and tocopherol were added in milk to enhance the flavor and photo-oxidative stability. It was done on fresh raw milk collected from Virginia Tech Dairy Farm through gas chromatography. Ascorbic acid and tocopherol (100 mg/kg of milk fat) supplemented samples revealed better flavor and photo-oxidative stability as compared to non-supplemented samples [116]. Ascorbic acid significantly inhibited the degradation of riboflavin in light-exposed milk, and antioxidant activities were mainly attributed to the scavenging effect on singlet oxygen [138]. A research was conducted to assess the impact of tocopherol and Vitamin C against atopy development in infants. Increased concentration of Vitamin C in breast milk which is water soluble decreases the risk of atopy in infants [139,140]. Ascorbic acid is extremely helpful for the infants, also plays a significant role during developmental stage in the production of neurotransmitters and synthesis of carnitine, and improves the absorption of iron, while in human and cow milk, its concentration is approximately 40 and 20 mg/l, respectively [141]. Oxidation of ascorbic acid depends on temperature, light, oxygen, and amount of catalysts. Vitamin is regarded as primary lipid-soluble antioxidant; main job of this vitamin is to protect the PUFAs and associated biochemical compounds from peroxidation. Among the tocopherols, α-tocopherol is regarded as more powerful antioxidant; antioxidant activity of β-, γ-, and δ-tocopherol is about 80-90% less than α-tocopherol [142]. γ-Tocopherol is of high functional value; it can trap the nitrogen oxide species. It helps the body to prevent cardiovascular diseases and cancers; concentration of Vitamin E in cow milk is about 0.9 mg/ml (Table-5) [143-145], and summer milk has higher concentration than winter milk. The concentration of vitamin in human milk ranges from 3 to 13 mg/ml [146]. Batool *et al*. [147] studied the impact of supplemented Vitamin E and selenium on oxidative stability of cheddar cheese which efficiently inhibited the LPO and raised the shelf stability.

**Table-5:** Mineral and vitamin content of cow and buffalo milk.

Minerals	Cow milk (mg/100 g)	Buffalo milk (mg/100 g)	Vitamins	Cow milk (mg/100 g)	Buffalo milk (mg/100 g)
Calcium	122	112	Vitamin A*	46	69
Phosphorus*	119	99	Vitamin E*	0.21	0.19
Potassium	152	92	Thiamine	0.05	0.05
Magnesium	12	8	Riboflavin	0.17	0.11
Sodium	58	35	Niacin	0.09	0.17
Zinc*	0.530	0.410	Pantothenic acid	0.37	0.15
Iron**	0.08	0.161	Vitamin B6	0.04	0.33
Copper**	0.06	0.035	Vitamin B12	0.45	0.40
Manganese	0.02	0.027	Biotin	2.00	13
Iodine	0.021	0.004	Vitamin C*	0.09	2.50
Se*	0.96	0.006	Vitamin D	2.00	2.00
References	[143]	[144]		[145]	[144]

* Chemical constituents that have antioxidant activity in milk.

** Chemical constituent that has pro-oxidant activity

## Antioxidant System

The antioxidant system of the human and animal body is comprised of two components: The antioxidant molecules and antioxidant enzymes.

### Antioxidant molecules

Antioxidant molecules have the capability of either accepting or donating an electron to terminate the unpaired state of a molecule. They are either endogenous or taken from exogenous sources through food, especially in milk. The endogenous antioxidant molecules are GSH, uric acid, ferritin, coenzyme Q, bilirubin, alpha lipoic acid, L-carnitine, and melatonin [148], while ascorbic acid (Vitamin C), retinoic acid (Vitamin A), and α-tocopherol (Vitamin E) are some of the familiar examples of antioxidant molecules taken from exogenous sources present in milk [149,150]. Some of the milk constituents have antioxidant properties as described above. In addition, many studies have shown that the antioxidant capacity of milk products can be enhanced (Table-6) through biotechnological approaches [25,56,151-167].

**Table-6:** Antioxidant characteristics of some dairy products.

Study	Result	References
Effect of grazing on antioxidant characteristics of sheep milk	Grazing improved the total antioxidant capacity of sheep milk	[151]
*Zingiber officinale* and *Beta vulgaris* were added in yogurt milk to improve the antioxidant capacity of buffalo, cow, and goat milk yogurt	Supplementation of yogurt milk with *Zingiber officinale* and *Beta vulgaris* improved the DPPH and ferric reducing antioxidant power	[152]
DPPH and ferric reducing antioxidant power assays were used to determine the antioxidant capacity of milk along with conventional methods such as peroxide value, thiobarbituric acid value, and loss of Vitamins A and E	DPPH and ferric reducing antioxidant power assays provided useful information regarding antioxidant capacity of milk	[153]
The antioxidant capacity of yogurt, *Lactobacillus acidophilus* milk, buttermilk, and vegetable-flavored fermented milk was analyzed for their antioxidant potential	The presence of probiotic *Lactobacillus casei* strains in the product positively improved the ferric reducing antioxidant power	[154]
The effect of cow feed supplementation by carrots on the β-carotene and α-tocopherol concentration in butter oil	Feed supplementation by carrots contributed in more stable β-carotene, as well as 30% higher α-tocopherol concentration (p<0.05)	[155]
The effect of betel leaf (*Piper betel* Linn.) extract on the physicochemical, sensory, and antioxidant properties of khoya made from cow milk and stored under room temperature	Khoa with 0.5 aqueous extract of betel leaves restricted the production of free fatty acid compared to control due to antioxidant property of betel leaves	[156]
The antioxidant properties of kefir produced from goat milk with kefir grains were investigated using total phenolic contents and DPPH assays	Antioxidant capacity of kefir was more than parent milk	[157]
Antioxidant properties of milk oligosaccharides from various ruminants were studied	The result suggests that milk oligosaccharides derived from certain ruminant species could be used as natural antioxidants	[158]
The effect of *Pediococcus pentosaceus* on antioxidant characteristics of probiotic yogurt was studied in cow, goat, and camel milk	Results evidence that antioxidant of goat milk yogurt was 93% as compared to 86% in camel milk. These results suggested that antioxidant characteristics of yogurt can be enhanced by probiotic bacteria	[56]
Cow milk was fermented by *Lactobacillus lactis* and *Lactobacillus delbrueckii*	Antioxidant capacity of milk fermented with *Lactobacillus, Lactobacillus lactis,* and *Lactobacillus delbrueckii*, was 21.91% and 29.7%	[159]
The effect of fish oil, Opal linseed, and Szafir linseed on the antioxidants of polish Holstein Friesian cow’s milk	Total antioxidative status increased in all experimental groups; however, the highest peak was recorded in fish oil+Szafir linseed and Szafir linseed group	[160]
Impact of *Lactobacillus delbrueckii* subsp. *bulgaricus, Lactobacillus rhamnosus*, *Streptococcus thermophilus* or *Lactobacillus delbrueckii* and *Lactobacillus fermentum* on antioxidant capacity of bovine milk and whey	Bacterial strains improved the DPPH free radical scavenging activity, inhibition of superoxide anions and lipid oxidation and reduces the atherogenesis in humans	[161]
Effect of supplementation of Pirotski Kachkaval by ethanolic extract of *Kitaibelia vitifolia* on antioxidant characteristics was investigated	Supplementation of Pirotski Kachkaval cheese by the ethanolic extract of *Kitaibelia vitifolia* raised the antioxidant capacity of cheese	[162]
Antioxidant characteristics of ice cream were increased by partially replacing the sucrose with sugarcane juice	Addition of sugarcane juice in ice cream increased the total phenolic contents, DPPH free radical scavenging activity, nitric oxide free radical scavenging activity, and total antioxidant capacity of ice cream	[163]
Interesterified blends of butter oil and *Moringa oleifera* oil were characterized for antioxidant capacity and storage stability	Phenolic compounds of *Moringa oleifera* oil enhanced the antioxidant perspectives and storage stability of butter oil in long-term storage	[164]
Peel extract was determined on antioxidant characteristics of whey butter	Addition of 400 ppm ethanolic extract of almond peel increased the total phenolic contents and DPPH free radical scavenging activity	[25]
Cheddar cheese was supplemented with mango (*Mangifera indica* L.) oil to improve the antioxidant characteristics	Supplementation of mango kernel oil increased the total phenolic contents, DPPH free radical scavenging activity, and nitric oxide free radical scavenging activity and inhibited the lipid oxidation	[165]
Influence of interesterified *Moringa oleifera* oil on oxidative stability of ice cream was studied	Addition of interesterified *Moringa oleifera* oil significantly improved the oxidative stability of ice cream	[165]
The main objective of this study was to raise the antioxidant characteristics of cheddar cheese of chia oil. Cheddar was supplemented with chia (*Salvia hispanica* L.) oil from 2.5% to 10%	Supplementation of cheddar cheese with chia oil increased the antioxidant capacity of cheddar cheese	[166]
Antioxidant characteristics of milk were enhanced by *Hypotrigona squamuligera* honey	Fortification of milk with *Hypotrigona squamuligera* honey inhibited DPPH free radicals with a lower peroxide value	[167]

DPPH=2,2-Diphenyl-1-picrylhydrazyl

### Enzymatic antioxidants

#### SOD

SOD catalyzes the removal of free O_2_^−^ and safeguards the cells against harmful effects through the following reaction.


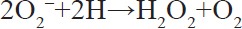


**Figure-3:**
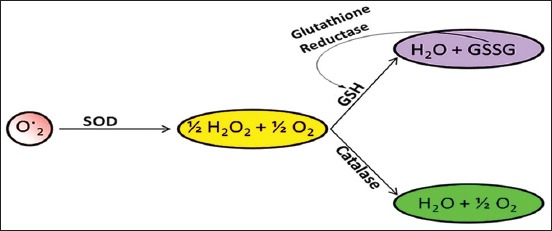
Endogenous antioxidant enzymes - superoxide dismutase, catalase, and glutathione antioxidant mechanisms of action [Figure designed by Mohammed Bule].

Catalase, GSHPx, or other reducing agents convert H_2_O_2_ to H_2_O, hydrogen peroxide formed from O_2_^−^, and oxidases are eliminated by catalases and peroxidases as shown in Figure-3 [60]. Cytosolic Cu/Zn-SOD, mitochondrial Mn-SOD, and extracellular-SOD are the major forms of SOD [168]. SOD can inhibit LPO. In cow milk, SOD is exclusively present in skim milk fraction, with a concentration of 0.15 mg/l-2.4 mg/l [57]. Human milk has 2.0-2.3 times more concentration of SOD as compared to cow milk.

#### GSHPx

GSHPx is a Se encompassing enzyme that provides protection against LPO, and it catalyzes the breakdown of _H_2_O_2 and organic hydroperoxides (R-OOH) by GSH (γGlu.Cys.Gly) as per the following chemical reaction [15].


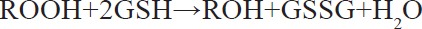


More than 90% of GSHPx exists in milk as an extracellular enzyme, it is the only enzyme which fixes Se (about 30% of the total), its concentration varies among the mammals, and concentrations are in the order: Human > caprine > bovine [169]. The concentration of GSHPx in cow milk ranges from 12 to 30 U/ml and its activity is mainly dependent on the concentration of Se. Antioxidant activity and Se content decrease with the progression of lactation [170].

#### GSH reductase

GSH reductase converts GSH disulfide to the sulfhydryl form (GSH), which then acts as a scavenger of free radicals as shown in Figure-3 [149]. It is present in a minute amount in the dairy milk for the production of antioxidant GSH in humans [171,172].

#### GSH transferases

There are three families of GSH transferases which are divided on the basis of location. They are cytosolic, mitochondrial, and membrane-associated microsomal. They help the body to neutralize secondary metabolites such as aldehydes, epoxides, and hydroperoxides [173,174]. This enzyme helps in the detoxification of toxicants and metabolites in the body. It inhibits the activities of kinase involved in the MAPK pathway that regulates cell proliferation and death [175,176].

#### Catalase

Milk catalase is a heme protein, molecular weight of catalase is 200 kDa, with isoelectric pH of 5.5, and this enzyme is stable in a wide range of pH 5-10 and rapidly loses activity when it is out of this pH range [169]. Most of the catalyzes contain heme, catalase causes the dismutation of H_2_O_2_, a chemical reaction in which H_2_O_2_ causes oxidation of the other H_2_O_2_ molecules, consequently, one is converted to O_2_, and the other two are converted to two molecules of H_2_O [177]. A polarographic method showed that average catalase activity in cow milk was 1.95 U/ml [178]. The concentration of catalase in human milk is approximately 10 times greater than cow milk [179].

## Milk Products and Their Antioxidant Activities

### Antioxidant characteristics of cheese

Cheese is one of the major fermented dairy products. Dairy products are an excellent source of high-quality protein and milk fat desirable for human health. It has fat-soluble vitamins along with vital mineral, calcium, and phosphorous and concentrated source of energy [180]. A study was performed in cottage cheese, a traditional Mexican Cheese, to investigate the antioxidant properties of peptides produced during the course of 6 months ripening period. Peptides were characterized by high-performance liquid chromatography (HPLC); results showed that peptides with antioxidant activity were produced during the ripening period of 6 months. 2,2-diphenyl-1-picrylhydrazyl (DPPH), free radical scavenging activity of 6-month-old cheese, was 98% [181]. Cheddar cheese was prepared using *Lactobacillus paracasei* as starter culture, changes in antioxidant characteristics of cheddar were monitored for 6 months, and different antioxidant assays were used as indicators of antioxidant activity. DPPH and superoxide free radical scavenging activities of cheese increased up to 4 months of ripening; the increase in antioxidant activities was attributed to the production of water-soluble peptides. Antioxidant activity and extent of water-soluble peptides were strongly correlated [182]. Antioxidant activity of white brined prepared from overheated milk (90°C, for 10 min) was investigated, antioxidant activity of water-soluble and water-insoluble fraction of cheese was increased during the ripening period, and antioxidant activities were correlated with degree of proteolysis [183]. Rashidinejad *et al*. [184] worked on full-fat cheeses to investigate the effect of antioxidant properties of green tea catechin. Full-fat cheeses were added with green tea extract (GTE) at 250, 500, and 1000 ppm. Cheeses were ripened for 90 days at 8°C. The compositional analysis of cheeses such as pH, total protein content (TPC), and antioxidant activity on storage days of 0, 30, and 90 was analyzed. The determination of GTE catechins from the ingesta and concentrations in the curd of ripened cheeses were assessed using HPLC. The resulted data showed that pH of whey and curd significantly decreased after addition of GTE during manufacturing of cheese and ripening period; however, no significant changes were recorded on fat, moisture, and protein contents of the cheese. The study further revealed that TPC and antioxidant activity also increased in all concentrations after GTE addition, but the manner was non-linear. Branciari *et al*. [185] studied pecorino cheese for total phenolic content and antioxidant activities after the supplementation of rosemary leaf in the diet of sheep. Two dietary groups were assigned after random collection of 324 sheep, which were kept on lucerne hay and concentrate (400 g/day) at recommended level. Thus, 2.50% dried rosemary leaves were supplemented to one group in the form of concentrate. The time period for the said study was 7 weeks. Cheese was prepared at 3, 5, and 7 weeks before the trial started. The supplementation of rosemary leaves showed good results because of the increase in the total phenolic content and antioxidant properties of the cheese and decreased lipid oxidation of pecorino cheese. Rashidinejad *et al*. [186] studied that the addition of catechin has no effect on low-fat hard cheese composition and increased total phenolic content and antioxidant activities. Cheese was analyzed for 90-day ripening period at temperature of 8°C [187]. According to this study, there was a significant increase in both the TPC and antioxidant activity during the ripening period of 90 days. Fernandes *et al*. [188] evaluated cream cheese oxidation and fermentative stabilities using oregano and rosemary essential oils which were added as a natural antioxidant in cream cheese. Peroxidase and anisidine value of treated cheese was estimated. Cheese treated with oregano and rosemary essential oils showed more stability, lower PV, and lower acidity with higher pH and also reduced the total viable count on 35 days of storage. The study showed that cheese treated with oregano had more acidic pH which is 4.68, less total viable count 2.35 CFU/g than rosemary essential oil [189]. Hence, protective effect of oregano and rosemary essential oils has been shown on cheese against lipid oxidation. The study was conducted using rosemary extract (RE). RE was added into the soft cheese, which was made from UF milk having 1.5% milk fat. RE was added in the concentration of 1-5%, and cheese was stored in cold storage for 30 days. The study showed that addition of RE had increased the total phenolic content and antioxidant capacity of cheese. Pasteurization had increased the total phenolic content and antioxidant capacity of cheese supplemented with RE. Addition of salt up to 3% had decreased the total phenolic content and antioxidant activity. The study showed that 1% RE had more total phenolic content and had more antioxidant activity, while 5% of RE tends to improve the nutritional value and had better acceptability, better flavor, texture, and more antioxidant activity during 30 days of storage [190]. Catechins were added in the low-fat hard cheese to evaluate its antioxidant and total phenolic content. Cheese was analyzed for 90 days of ripening at 8°C. Cheese curd showed retention of catechins in the range of 0.63-0.75. Catechins decreased the pH of cheese and did not affect protein, fat, and moisture content of the cheese. During 90 days of ripening, both total phenolic content and antioxidant activities were increased [186]. *Foeniculum vulgare* (Fennel) was added into the cottage cheese. The extract is rich in the phenolic content. Fennel antioxidant and antimicrobial potential were determined. The study showed that fennel extract has phenolic compounds in it. Fennel was added in the cottage cheese, to increase the shelf life of the cottage. In control cheese (where no fennel was added), no effects on nutritional profile up to 7 days of storage were observed. After 14 days of storage, it showed a decrease in the lactose content in treated cottage cheese. Only control cheese showed the signs of deterioration after 14 days. Fennel (phenolic-enriched extract) cottage cheese increased the shelf life up to 14 days through its antioxidant property [93]. Another study was carried out on four different plant essential oils (bay, cinnamon, clove, and thyme), which were added in the soft cheese in low-fat and full-fat cheese as a natural preservative. The plant essential oils were added at the concentration of 0.1, 0.5, and 1%, and its antimicrobial effect was evaluated on *Listeria monocytogenes* and Salmonella for 14 days at two different 4°C and 10°C storage temperatures. All these oils at 1% concentration were equally effective in reducing the activity of *Listeria* in low-fat cheese. However, in case of full-fat cheese, only clove oil achieved this reduction. The study also showed that thyme oil has no effect on Salmonella in full-fat cheese [191]. *Matricaria recutita* L. (chamomile) was added into the cottage cheese as a natural antioxidant and to also improve its nutritional value. The study showed that the antioxidant activity was limited for 7 days only. The aqueous plant extract has microencapsulation, so it is added to the cottage cheese to increase the bioactivity. Cottage cheese functionalized with chamomile extract showed a higher value of antioxidant activity for 7 days only, but it did not affect the nutritional value of cottage cheese [93]. The study was conducted to check the antimicrobial and nutritional effect of *Moringa oleifera* leaf ethanolic and ether extracts which were added into the West African soft cheese in different concentration (1, 2, and 3%). The study showed that the highest antimicrobial effect was shown by the 2 and 3% of ethanol extract of *M. oleifera*. The addition of 1% ethanol showed the highest crude protein and moisture. As compared to ether extract, ethanol extract tends to improve nutritional and microbial safety of West African soft cheese [192].

### Antioxidant characteristics of yogurt

The effect of *Pediococcus pentosaceus* on antioxidant characteristics of probiotic yogurt was studied in cow, goat, and camel milk, and the results showed that antioxidant of goat milk yogurt was 93% as compared to 86% in camel milk. These results suggested that antioxidant characteristics of yogurt can be enhanced by probiotic bacteria [193]. Yogurt is a fermented milk product with distinctive therapeutic value, presented in diversified forms and flavors. Yogurt was added with carrots, pumpkin, broccoli, and red sweet pepper at 10% concentration, and ferric reducing antioxidant power (FRAP), and DPPH assays were used for antioxidant activity during the storage period of 14 days. Yogurt added with broccoli, and red sweet pepper revealed higher DPPH free radical scavenging activity and FRAP. However, antioxidant activity decreased during the storage period of 14 days [154]. Cow, buffalo, and goat milk yogurts were supplemented with aqueous extracts of *Zingiber officinale* and *Beta vulgaris*. DPPH free radical scavenging activity and FRAP of goat milk yogurt were greater than other cow and buffalo milk [152]. In another study, antioxidant capacity of yogurt was increased by supplementing the yogurt milk with 60 mg Vitamin C, 12 mg Vitamin E, and 3 mg β-carotene. Antioxidant characteristics of supplemented yogurt were higher than non-supplemented yogurts with no effects on sensory properties [194]. Yogurt was supplemented with fruit pulp of papaya and cactus pear using *Lactobacillus bulgaricus* and *Streptococcus thermophilus* as starter cultures, and total phenolic contents, ascorbic acid, and total antioxidant activity were analyzed. Yogurt added with papaya fruit pulp had higher total phenolic contents, antioxidant activity, and Vitamin C concentration [195].

## Oxidative Stability of Milk and Milk Products

Dairy industry is facing a concern regarding the oxidative stability of milk and dairy products. The negative impact of oxidation reactions in milk is related to strong off-flavors and deterioration of nutritional quality in milk. There is a delicate balance between anti- and pro-oxidative processes of milk and dairy products. Oxidative stability of milk and dairy foodstuffs depend on fatty acid structure, contamination with metal ions, and concentration of tocopherols and carotenoids [196]. Processing, packaging, storage conditions, and period have pronounced effect on the extent of natural antioxidants, which is directly connected with oxidative stability of pasteurized milk and dairy products [197,198]. It is extremely important to determine the antioxidant capacity of milk and milk products (Table-7), as oxidation can only happen when there is a difference between the ROS and the antioxidants defense mechanism [199]. Antioxidant activity is used to monitor the oxidation in a product. Antioxidant capacity of milk and milk products is mainly attributed to tyrosine and cysteine, Vitamins A and E, carotenoids, and enzyme systems such as SOD, catalase, and GSHPx [200]. Equol, a polyphenolic metabolite of daidzein, is also present in milk in a significant amount [58]. Therefore, it is extremely important to determine the total antioxidant capacity of milk and its products throughout various intervals.

**Table-7:** Relative rates (M^−1^S^−1^) of oxidation by triplet (autoxidation) and singlet (photo-oxidation) oxygen.

Oxygen	Oleic acid (C18:1)	LA (Ac18:2)	LA (C18:3)
Triplet O_3_	1	27	77
Singlet O_2_	3×10^4^	4×10^4^	7×10^4^

LA=Linoleic acid

Antioxidant capacity assays are important for general antioxidant activity in foods and can be categorized into two main assays: Hydrogen atom transfer-based assays and electron transfer-based assays [201]. Oxygen radical absorbance capacity reported by Benzie and Devaki [63] shows that hydrogen atom transfer-based assays evaluated antioxidant activity from amino acids in milk that can act as hydrogen donors. Determination of nitric oxide free radicals, total phenolic contents, flavonoid contents, DPPH free radicals, inhibition of oxidation of linolenic acid, and total reducing capacity can be used for the characterization of antioxidant capacity in milk and dairy products [202]. Lipid oxidation in milk can be measured by several methods; these include instrumental methods, such as transition in fatty acid profile, concentration of Vitamins A, E, and C, and total antioxidant assays. Peroxide value measures the primary stages of autoxidation; it is a useful parameter to determine the oxidation status of milk, cheese, butter, and ice cream [203,204]. TBA test is also used for the estimation of secondary oxidation in food products. Nadeem *et al*. [205] demonstrated that the use of olein fractions improves the oxidation status of ice cream. Sensory techniques are also commonly used for the assessment of oxidized flavor in milk and milk products [206].

### Use of metabolic modifiers in food animals

Metabolic modifiers are compounds that may alter metabolism to improve the efficiency of productive processes, such as growth rate, milk yield, body composition, or nutrient utilization. Extensive research and several studies have been done on metabolic modifier compounds in animal and poultry industries. These compounds can be divided into several categories.

- Antibiotics and organic arsenic compounds as growth promoters or feed additives.
- Anabolic compounds (progesterone and related compounds), which may stimulate protein and amino acids metabolism.
- Estrogens and other related compounds play an important role in increasing fat deposition and improving fattening of poultry and animal.
- Thyroxin and related compounds may improve the metabolic rate and growth performance.
- Thyroid depressants such as thiouracil and thiourea may increase fat deposition.

Only a few such hormone-like compounds are permissible feed additives. Certain possible additives are either effective for the intended purpose, or the conditions for use are very critical in general. Others leave some residues in edible organs, which make them unacceptable.

Metabolic modifiers such as antibiotics can affect the growth and digestion as well as digestive tract efficiency by affecting microbial population. Other substances, such as alpha-adrenergic agonists, anabolic steroids, and somatotropins, may disturb breakdown and usage of fascinated bioactive nutritive compounds [207]. Growth hormones are proteins that are usually injected into animals to promote growth. At present, hormones are used by a large number of beef and dairy farmers to get more products with fewer animals. To date, beef cattle are the only meat-producing animals that are approved for receiving hormone injection in the United States and Europe [208,209]. The hormones can be administered through an implant under the skin of the ear that delivers measured amounts of the hormone throughout the animal’s life, and such animals convert feed into muscle faster and more efficiently [210]. The meat produced by this method is also lean than conventionally grown animals. Bovine somatotropin (BST) or recombinant bovine growth hormone is the only growth hormone approved for use in dairy cattle use. The hormone is administered through injection and simulates certain natural hormone such as insulin-like growth factor-1, which helps the cows to convert nutrients into milk, thus cows administered with BST have higher milk production than non-injected cow [211]. On the other hand, the use of antibiotics in food animal is a complex issue that is quite often oversimplified by both critics and proponents. It has evoked lots of concern about antimicrobial resistance. The estimated farm-to-fork risks from farm antibiotic use are extremely low. The meat or milk from an animal undergoing antibiotic therapy is allowed to enter the food supply only after the withdrawal period has passed, and the animal’s system has been given time to sufficiently clear the medicine. The Food and Drug Administration (FDA) has documented required periods for withdrawing medication for each drug and species. However, easy access to antibiotics is required for the prevention and treatment of animal diseases and production of safe food. Therefore, policy actions regarding the use of antibiotics in food animal production should be based on scientific knowledge after carefully weighing the consequences of any specific course of action [212].

### Potential benefits of functional foods of animal origin

There are several encouraging facts for the use of functional foods of animal origin. The major reason would be that these products can serve nutritious food to the growing population particularly in the developing countries [213]. The use of hormones such as BST has led to increase in the output of milk from cow, increasing the income of farmers. From the consumers’ point of view, BST has no risk on the health, and it gets degraded in the body since it is a protein [214]. Transgenic technology can be used to produce milk and egg with less fat and more vitamins and minerals, which are essential. Similarly, as per consumers’ need, it is possible to incorporate the specific nutrients in functional foods [214]. Animal pharming helps in the synthesis of pharmaceuticals from milk and meat [215].

### Main concerns of functional foods

Functional food products, along with its beneficial health effects, also possess some health-related hazards. GM foods are the main concern of public health in terms of environmental safety, labeling, intellectual property rights, food security, and poverty alleviation and consumer choices. Most authorities such as European Commission, India Parliamentary Committee of Agriculture, and FDA consider that specific assessments are necessary for GM food and there is a lack of policies regarding safety testing of foods, regulations, and labeling. All the novel methods and concepts should be well defined which are used for the assessment of GM and conventional foods [216]. The potent issues or health concerns have increased using these foods and the privileges are by changing its chemical nature through biotechnological approaches which disturb the nutritional profile, drug resistance for certain foods, potential toxin production in the functional foods, allergenicity, possible creation of new viruses and toxins, religious/cultural/ethical concerns, concerns of animal rights groups, and fear of the unknown issues [217,218]. For example, the risks behind recommending the increased dose of chemical compounds such as “isoflavones” may be dangerous because of modulation in the estrogen metabolism and the development of different kinds of tumors in clinical research animals due to genistein, which is a soy phytoestrogen and may act as a “double-edged sword” [219]. The International Food Information Council released a series of the recommendations that are expected to guarantee that research results come about nutritional status, safety of foods, and health risks are conveyed in an unmistakable, balanced, and non-deceiving way [220]. The following recommendations have been made by the General Accountability Office related to the safety of functional foods.

- Develop and proclaim regulations or other directions for industry on the proof expected to document, which satisfy the safety and use of new dietary ingredients in dietary supplements.
- Develop and proclaim regulations or other dietary guidance for industry on the safety-related information required on labels for dietary supplements and functional foods.
- Develop a superior database analytical system for recording and analyzing reports of functional foods and dietary supplements and their potent health problems [221].

### Functional foods from consumers’ point of view

Functional foods are mainly developed to provide people with good/high-quality foods for their healthier life, but consumers around the world have different views. Consumers’ views on functional foods have been collected from different countries during different periods, which indicate that views change among people around the globe [222]. Research results indicate that people from America are more informative about the functional food and they are ready to accept these foods into their regular diet chart. People in Europe are different from Americans, and they doubt about these novel functional foods [223]. An online poll conducted in the countries of European Union named Eurobarometer opinion poll showed that there was either weak support or opposition for these GM food and crops. Compared to earlier polls, there is an increase in opposition against these GM food and crops [224]. Danish people consider the designer food as impure and not natural. In Finland, the scenario is different as the consumers have a good response to designer foods [225]. Taste of food plays a major role among selection of food by the consumer, and hence, the taste of functional food remains the same even after fortification with various ingredients [226]. Another study on gender preference for functional food reports that females buy more functional food than male [227]. Studies in the UK came with a conclusion that people who are educated tend to seek functional food either to reduce their body weight or reduce cholesterol level [228]. People all over the world are mostly new to the concept of designer food/functional food/genetically modified food, and hence, media play a crucial role in spreading good or bad news about these products. Media should focus on the positive points of the functional food so that people are motivated to accept designer/functional food, which can serve nutritious food to the starving community in the developing countries.

## Conclusion and Future Perspectives

It has been concluded that functional foods such as milk and its products have antioxidant activities which may enhance human health, immunity, nutritive values, prevent or treat health threats, help in fighting some diseases such as diabetes and cancers, reduce blood pressure and cholesterol-related problems as well as ameliorate cardiovascular disorders, lower risk of chronic diseases, and allergic reactions. In particular, free radicals such as ROS and RNS generated during normal cellular, and biochemical reactions play a major role in the pathogenesis of the chronic diseases. The free radicals are usually counteracted by body’s own antioxidant system. However, in certain conditions, either the body’s defense mechanism will be weak, or the production of free radicals will outweigh. Therefore, controlling the pro- and anti-oxidant balance through antioxidant-enriched diet is important. Designer foods such as milk can be used against lifestyle disorders due to the presence of antioxidant activities. In the new era, functional or designer foods are still new. The development of genetically modified animals for food and industries is one of the most promising applications of biotechnology, but all the claims surrounding functional food health promoting benefits must be backed by a good scientific background. Genetic engineering has a great influence on the production of functional as well as designer foods which, in turn, is a great addition to the agriculture, livestock, pharmaceutical, and socioeconomic sector. Milk and related dairy products are gaining some attention due to their extraordinary proprieties which will have a direct influence on the health concerns, economic status, and environmental status that can cover all the aspects on benefits relative to risk which should be analyzed before commercial production. Research is a necessary tool to explore the benefits of functional foods which can satisfy both consumers and producers through verbal communication. Improved health benefits, enhanced profitability of products produced by the novel scientific techniques, and social and ethical issues, such as animal welfare, environmental impact, regulatory processes, and safety, all contribute to the acceptance of the “designer” products. All these points need to be addressed well in advance before commercialization and introduction of the designer food for human consumption. Although there are various advantages of GM of food, there remain some limitations that need to be addressed immediately so that customer/consumers can be convinced to accept these products. Safety of these food designed by genetic manipulation needs to be addressed, and toxicity studies and flavor studies should also be conducted.

## Authors’ Contributions

MN gave the idea and prepared the outlines. MB designed the figures along with comprehensive oxidative stress portion writing. ITK, RU, and SA contributed equally in the drafting of the manuscript, while KN wrote enzymatic antioxidant portion and did critical editing and reviewing of the whole manuscript. All the authors have read and approved the final version.
